# Analysis of Gene Expression Profiles, Cytokines, and Bacterial Loads Relevant to Alcoholic Liver Disease Mice Infected With *V. vulnificus*

**DOI:** 10.3389/fimmu.2021.695491

**Published:** 2021-08-20

**Authors:** Zi-Han Feng, Shi-Qing Li, Jia-Xin Zhang, Bin Ni, Xin-Ru Bai, Jian-Hao Xu, Zhen-Bo Liu, Wen-Wen Xin, Lin Kang, Shan Gao, Jing Wang, Yan-Wei Li, Jia-Xin Li, Yuan Yuan, Jing-Lin Wang

**Affiliations:** ^1^State Key Laboratory of Pathogen and Biosecurity, Beijing Institute of Microbiology and Epidemiology, Academy of Military Medical Sciences (AMMS), Beijing, China; ^2^School of Medicine, Jiangsu University, Zhenjiang, China; ^3^College of Life Sciences, Hebei Normal University, Shijiazhuang, China; ^4^School of Life Sciences, Fujian Agriculture and Forestry University, Fuzhou, China; ^5^Rongcheng International Travel Health Care Center, Rong Cheng Customs, Rongcheng, China

**Keywords:** *Vibrio vulnificus*, alcoholic liver disease, RNA-seq, cytokines, bacterial loads

## Abstract

Patients with liver disease are susceptible to infection with *Vibrio vulnificus* (*V. vulnificus*), but the specific reasons remain elusive. Through RNA-seq, we found that when mice with alcoholic liver disease (ALD) were infected with *V. vulnificus* by gavage, compared with the Pair group, the small intestinal genes affecting intestinal permeability were upregulated; and the number of differentially expressed genes related to immune functions (e.g., such as cell chemotaxis, leukocyte differentiation, and neutrophil degranulation) decreased in the liver, spleen, and blood. Further analysis showed that the number of white blood cells decreased in the Pair group, whereas those in the ALD mice did not change significantly. Interestingly, the blood bacterial load in the ALD mice was about 100 times higher than that of the Pair group. After the ALD mice were infected with *V. vulnificus*, the concentrations of T cell proliferation-promoting cytokines (IL-2, IL-23) decreased. Therefore, unlike the Pair group, ALD mice had weaker immune responses, lower T cell proliferation-promoting cytokines, and higher bacterial loads post-infection, possibly increasing their susceptibility to *V. vulnificus* infection. These new findings we presented here may help to advance the current understanding of the reasons why patients with liver disease are susceptible to *V. vulnificus* infection and provides potential targets for further investigation in the context of treatment options for *V. vulnificus* sepsis in liver disease patient.

## Introduction

*Vibrio vulnificus* (*V. vulnificus*), a Gram-negative aquatic bacterium, can cause severe gastroenteritis from raw seafood consumption, with sepsis-related mortality rates of 50% (1). Although infections are rare, this pathogen is responsible for over 95% of seafood-related deaths, and carries the highest fatality rate of any food-borne pathogen in the USA (2). In addition, peer-reviewed reports on *V. vulnificus* infections in Europe, China, South America, and Japan have been published and showed that human infections usually occurred following the consumption of contaminated seafood, which can cause gastroenteritis and septicemia (3–7). Global warming and rising sea temperatures have caused the geographic area affected by *V. vulnificus* to expand into previously unaffected areas, suggesting that infections may increase in future (8). Almost all *V. vulnificus* cases occur in patients with underlying diseases (9). The most common risk factors are liver disease and diabetes (2). In a case series of 181 patients reported by Shapiro et al. (1998), chronic liver disease was present in 80% of the patients and, even more strikingly, 61% of the fatal cases had an underlying chronic liver disease, of which, 74% had cirrhosis or alcoholic liver disease (ALD) and 24% had hepatitis (10). Bross et al. (2007) reported that 97% of patients have some form of chronic disease, including liver disease (80%), alcoholism (65%), and diabetes (35%) (11). Thus, liver disease, especially ALD, is a major susceptible factor for *V. vulnificus* infection.

Many researchers believe that elevated serum iron levels is the main reason why ALD patients are susceptible to *V. vulnificus* infection, since iron has been shown in numerous studies to be critical for the survival and growth of *V. vulnificus* (12, 13). Researchers have used the inoculated iron dextran-treated mouse model to indicate that high serum iron levels can increase the pathogenicity of *V. vulnificus* (14). However, the serum iron concentration in many patients with liver disease is not significantly elevated (15); it is, in fact, much lower than the corresponding serum iron concentration in animal models (14). Therefore, elevated serum iron concentrations may not be the only factor causing ALD patients to be susceptible to *V. vulnificus* infection. It is important to acknowledge that in addition to being susceptible to *V. vulnificus* infection, ALD patients are also susceptible to infections with other microorganisms (16). Hence, the reason for their susceptibility may be related to other common factors such as the immune status of the patient. Compared with the blood of healthy people, the activity of blood neutrophils in patients with chronic liver disease is reduced and the complement content is lower, which favors *V. vulnificus* and *Escherichia coli* survival in the blood (17). In addition to neutrophils, macrophages have also been suggested to play a part in the defense against *V. vulnificus* (18, 19). Although there are many clinical studies on liver disease with *V. vulnificus* infections, there are a few systematic studies in animal models. Therefore, it remains unclear if the above factors differ in ALD patients and healthy people. It is also unclear which factors affect the susceptibility of ALD patients to *V. vulnificus* infection.

To explore why ALD patients are susceptible to *V. vulnificus* infection, we established a C57 mouse ALD model based on the NIAAA model (20), and simulated food-borne infection by gavage with *V. vulnificus* YJ016, a clinical strain isolated from patients with sepsis (21). At 12 hours post-infection when the *V. vulnificus* had spread from the intestine (22), we collected the mouse small intestine, liver, spleen, and whole blood samples for RNA-seq analysis; calculated the blood bacterial loads and cell counts; and assessed the inflammatory factors and other indicators. Unlike the Pair group, we found that ALD mice had weaker immune responses, lower T cell proliferation-promoting cytokines, and higher bacterial loads post-infection, possibly increasing their susceptibility to *V. vulnificus* infection. These new findings we present here may help to advance the current understanding of the reasons why patients with liver disease are susceptible to *V. vulnificus* infection and provide potential targets for further investigation in the context of treatment options for *V. vulnificus* sepsis in liver disease patients.

## Materials and Methods

### Ethics Statement

All animals were kept in a specific pathogen-free (SPF) environment. Procedures for care and use of the animals were approved by the Ethics Committee of Academy of Military Medical Sciences (Ethics Board approval number: IACUC-DWZX-2020-008). All applicable institutional and governmental regulations concerning the ethical use of animals were followed.

### Bacterial Strains and Culture Conditions

*V. vulnificus* YJ016 was isolated from patients with sepsis (21), which was sub-cultured in solid medium (Columbia blood or LBS agar plates containing 20 μg/ml polymyxin B) at 37°C. Before being used for gavage, YJ016 was grown to log phase in Luria-Bertani salt (LBS) medium (37°C, 150 rpm with shaking).

### Animals and Experimental Approach

**Figure 1:**
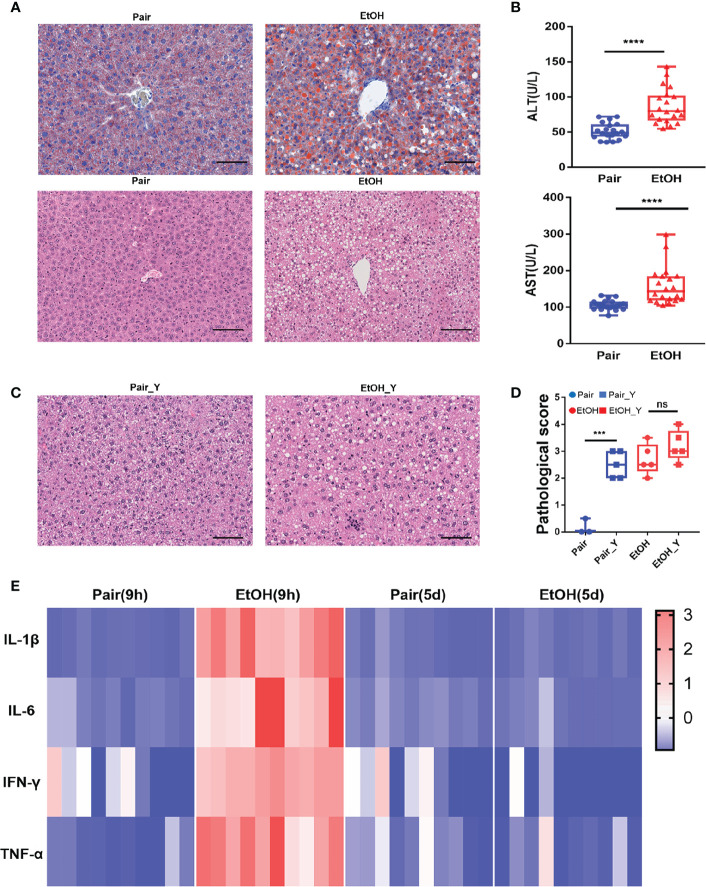
Evaluating the mouse model of ALD. **(A)** Representative oil red O and H&E staining in the liver tissues from the Pair and EtOH groups. Scale bars, 100 μm. There are round vacuoles of varying sizes, which are lipid droplets formed by fat accumulation in the liver cells of the EtOH group. **(B)** Serum ALT and AST levels (U/L) in mice from the EtOH group or the Pair group 9 hours post alcohol or maltodextrin gavage, respectively. (n = 20, ***P < 0.001, ****P < 0.0001). **(C)** Representative H&E in the liver tissues from mice in the Pair_Y and EtOH_Y groups. **(D)** Pathological scores of liver tissues in the Pair, EtOH, Pair_Y, and EtOH_Y groups. **(E)** Cytokine concentrations in plasma 9 hours and 5 days post gavage (n = 10). The concentrations of cytokines were standardized by z-score, with red indicating a higher concentration and blue indicating a lower concentration. ns, no significant.

The NIAAA model was improved upon to establish a mouse ALD model, as shown in **Figure 1A**. Nine-week-old female C57BL/6 mice (Beijing Vital River Laboratory Animal Technology Co.) were randomly divided into ethanol (EtOH)-fed group (n = 35) and Pair-fed group (n = 30). The EtOH-fed group received a 5% Lieber-DeCarli-EtOH (wt/vol) alcohol liquid diet (Lieber, Dyets, Inc.) for 15 days. The Pair group received non-EtOH liquid diets (Lieber-C), containing the same number of calories as the EtOH group. To adapt to the liquid diets, all the mice were fed a non-EtOH liquid diet for 5 days before being fed an EtOH or non-EtOH liquid diet. On day 10, the EtOH group was given 31.5% (vol/vol) ethanol and the Pair group was given 45% (wt/vol) maltodextrin by gavage. The volume (µl) of both was equal to the weight of the mouse (g) multiplied by 20. At 9 hours post gavage, 100 µl of blood was collected from 20 mice in each group through the tail vein; the serum was removed from each sample. Alanine Transaminase Activity Assay Kit (ab105134) and Aspartate Aminotransferase Activity Assay Kit (ab105135) were used to detect the concentrations of serum alanine transaminase (ALT) and aspartate aminotransferase (AST). The mice were then fed for another 5 days. On day 15, the two groups of mice without blood sampling (n = 10) were divided into two subgroups (n = 5), and then given 300 µl of LBS or YJ016 (~10^8^ colony forming units; CFU) by gavage. After 12 h of fasting, the blood and tissue samples of the liver, small intestine, and spleen were stored in TRIzol (Invitrogen) and quickly frozen in liquid nitrogen. Twenty mice in the EtOH and Pair groups with blood drawn from their tail veins, which were divided into two subgroups (n = 5), were given 300 µl of LBS or YJ016 (~108 CFU) by gavage. At 12 h post fasting, the livers were taken for hematoxylin and eosin (HE) staining and red oil O staining to evaluate the construction of the model. For H&E staining, the liver pathology scores were performed by the degree of hepatocyte steatosis and vacuolar degeneration (23). Whole blood (anticoagulated with EDTA) was taken from each mouse *via* eyeball, 150 µl of which was taken for bacterial and blood counts. The remaining blood was centrifuged at 3,000 × g for 15 min to separate the plasma and stored frozen at -80°C for further measurement.

### Sample Collection and Sequencing

RNA integrity was assessed using the RNA Nano 6000 Assay Kit for the Bioanalyzer 2100 system (Agilent Technologies, CA, USA), the library preparations were sequenced on the Illumina Nova seq platform and 150 bp paired-end reads were generated (Novogene Experimental Department). Raw reads of FASTQ format were firstly processed through in-house Perl scripts. In this step, clean reads were obtained by removing the reads containing adapter, poly-N, and low quality from the raw data. The index of the reference genome was built using the Hisat2 v2.0.5 (http://daehwankimlab. github.io/hisat2), and the clean paired-end reads were aligned to the reference genome using the same software (24). Feature Counts v1.5.0-p3 (http://subread.sourceforge.net/) was used to count the read numbers mapped to each gene of mouse (25). Default parameters were used in all software.

### Differential Expression Analysis and Enrichment Analysis

Differential expression analysis on four groups of samples (small intestine, liver, spleen, and blood) was performed using the DESeq2 R package v1.20.0 (http://bioconductor.org/packages/release/bioc/html/DESeq2.html) (26). The resulting P-values were adjusted using the Benjamini and Hochberg’s approach. Genes with adjusted P-values < 0.05 found by DESeq2 were assigned as differentially expressed genes (DEGs). Gene Ontology (GO), Kyoto Encyclopedia of Genes and Genomes (KEGG), and Reactome enrichment analysis of DEGs was implemented by the clusterProfiler v3.8.1 (https://yulab-smu.github.io/clusterProfiler-book/chapter5.html) (27). For the small intestine and blood samples, the number of DEGs was below 100 when using the adjusted P-values < 0.05, so genes with P-values < 0.05 and |log2Fold change| > 1 found by DESeq2 were assigned as differentially expressed in the enrichment analysis. Default parameters were used in all software.

### Real-Time Quantitative PCR (RT-PCR)

RT-PCR was conducted using the qTOWER 2.2 Quantitative Real-Time PCR Thermal Cyclers (Germany). First-strand cDNA was synthesized using the TUREscript 1st Stand cDNA SYNTHESIS Kit (Aidlab) according to the instructions of the manufacturer. Relevant information is provided in **Supplementary Table 1**. Detection was based on SYBR-Green I fluorescence. Thermal cycling conditions were 95°C for 3 min, followed by 40 cycles of 10 s at 95°C, 30 s at 58°C, 20 s at 72°C, and 60–95°C, +1°C/cycle, and a holding time of 4 s for the melting curve analysis. Gene expression was normalized to that of *β-actin*. The 2^−ΔΔCt^ method was used to calculate the relative gene expression levels.

### Blood Bacterial Load and Blood Count

Whole blood (100 µl) was diluted 10-fold in four gradients, and 100 µl of each gradient was evenly spread on LBS agar medium (containing 20 µg/ml polymyxin B). After 24 h of incubation at 37°C, the clones on the plate were counted. Blood counts were performed using the PET-6800 VET animal automatic blood cell counter (Shenzhen Prokan Electronics Co., Ltd.).

### Plasma Complement C3, C5, and Cytokine Detection

Plasma Complement C3 and C5 concentrations were measured by ELISA using the Complement C3 Mouse ELISA Kit (ab157711) and the Complement C5 Mouse ELISA Kit (ab264609), respectively. Plasma from the mice with LBS gavage in the Pair groups (n = 10) and EtOH groups (n = 10) were thawed and then tested according to operate as the instructions in the handbook. The Procarta Plex TM immunoassay kit (EPX170-26087-901) was used to measure the plasma cytokine concentrations. The following target cytokines were in 17-Plex immunoassay: IL-12p70, IL-13, IL-1β, IL-2, IL-4, IL-5, IL-6, TNF-α, IFN-γ, GM-CSF, IL-18, IL-10, IL-17A, IL-22, IL-23, IL-27, and IL-9. All samples included a repeat, and the average values were calculated.

### Statistical Analysis

All data were graphed and analyzed using the GraphPad Prism 7.04. Paired t-test was used to compare the number of DEGs. Pearson’s test was applied to analyze the correlation between RT-PCR and RNA-Seq results. Mann-Whitney test was used to compare the blood bacterial load. Unpaired t-test was used to compare complement C3 and C5. Two-way ANOVA was used to compare the differences between the white blood cells (WBC) and cytokines.

## Results

### Alcoholic Liver Injury of Mice in Different Groups

To explore the underlying mechanisms of ALD patients who are susceptible to *V. vulnificus* infection, we performed the optimized NIAAA model, using alcohol fed mice (EtOH group) and non-alcohol fed mice (Pair group). The mock-infected mice of Pair and EtOH group (LBS gavage) were named as “Pair” and “EtOH”, respectively. Similarly, the *Vibrio vulnificus*-infected mice in the Pair and EtOH groups (YJ016 gavage) were named as “Pair_Y” and “EtOH_Y”, respectively.

**Figure 2:**
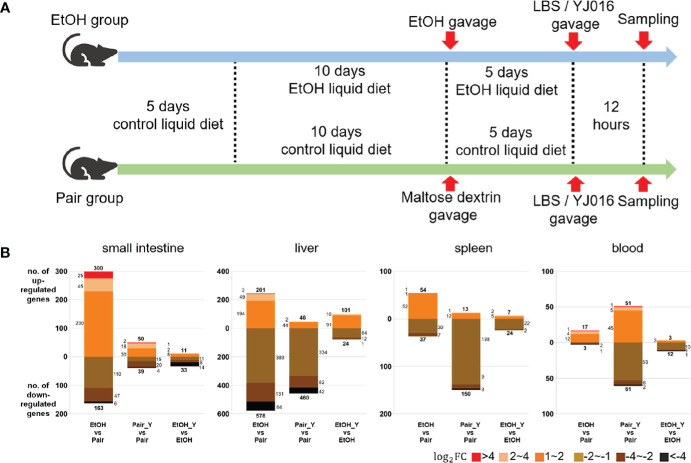
Experimental design and transcriptome response in mice after YJ016 infection. **(A)** On the fifth day after gavage (the 15th day of alcohol feeding), mice were given 10^8^ CFU *V. vulnificus* YJ016 culture or LBS as a control by gavage (n = 15 for the Pair and EtOH groups). After LBS or YJ016 gavage, five mice in each subgroup were used for RNA sequencing; The others were used for further analysis. **(B)** Transcriptome response of mice in the EtOH and Pair groups after infection with YJ016.

After the EtOH-fed for 15 days, oil red O staining showed that fat accumulated in the cells and formed red lipid droplets in the livers, and H&E staining showed that there were round vacuoles of varying sizes in the livers of the EtOH group (**Figure 1A**). At 9 h post EtOH gavage, the serum ALT and AST level (U/L) of the EtOH group was higher than that of the Pair group (**Figure 1B**), and the IL-1β, IL-6, TNF-α, and IFN-γ cytokines levels of the EtOH group had also increased significantly (**Figure 1E**). At this time, the acute inflammation caused by alcohol was dominant, which could affect the observation of the pathogenic process of bacteria. Therefore, we continued to feed the mice for another 5 days to avoid any interference from the alcohol gavage (**Figure 2A**) and most cytokines levels were restored to no significant difference compared with the Pair group (**Figure 1E** and **Supplementary Figure 1**).

The liver damage caused by *V. vulnificus* infection in the mice was previously reported (28, 29). In addition to the steatosis caused by ALD, we also found obvious vacuolar degenerations in livers of the Pair_Y group (**Figure 1C**). The pathological scores of the mice in the Pair and EtOH groups both increased after YJ016 infection (**Figure 1D**). However, there was no significant difference between the EtOH and EtOH_Y groups (**Figure 1D**), which may be due to the serious pathological damage by ALD.

### Quality Control of the Mouse Transcriptome

An average yield of 45.12 million raw reads was obtained from sequencing a single sample from each of the 80 libraries and 42.38 million clean reads were read into the mouse transcriptome on average. The total mapping rate was 96.89%–97.38%. The Q_20_ of the clean data was 97.66%–98.67%, and the Q30 was 93.17%–95.95%. The ratio of unique mapped reads to total mapped reads was 39.18%–51.29% for the blood samples, 89.06%–91.57% for the small intestine samples, 88.28–91.74% for the liver samples, and 83.0%–92.57% for the spleen samples. The read summary of the sequences is provided in **Supplementary Table 2**.

### Transcriptome Response of Mice in the EtOH and Pair Groups after Infection With YJ016

The number of DEGs between the “EtOH_Y” and “EtOH” was less than that between the “Pair_Y” and “Pair” in the small intestine, liver, spleen, and blood of the mice (**Figure 2B**), the detailed data was provided in Document S1. It implied that the mice in the Pair group might have a stronger transcriptome response after YJ016 infection than the EtOH group. It was worth noting that among the genes that changed more than twice after YJ016 infection, the number of downregulated genes was far more than that of the upregulated genes. A similar result was reported for *Pseudomonas plecoglossicida* and *V. parahaemolyticus* infections (30, 31), which showed that the number of genes that was downregulated in the host was higher than the number of genes that was upregulated in the early stages of infection. However, the downregulation of gene expression was not a proxy for a weakened function, so we conducted further studies through gene enrichment analysis and the specific function of the DEGs.

### Enrichment Analysis of the Immune-Related DEGs Between the EtOH and Pair Groups

**Figure 3:**
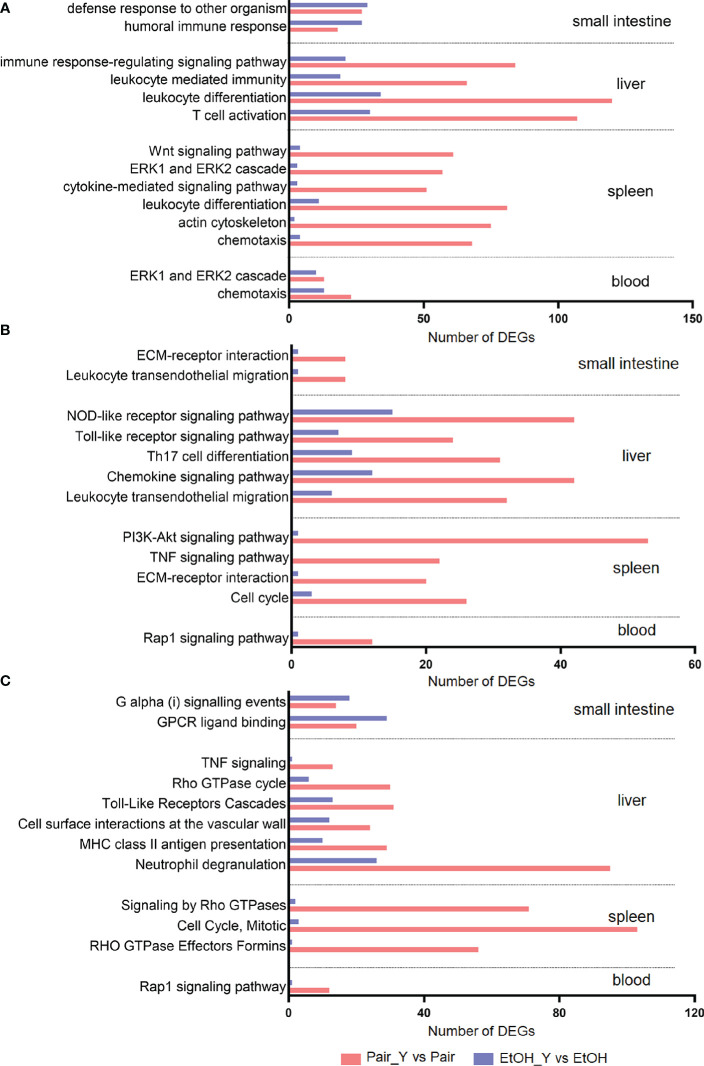
GO **(A)**, KEGG **(B)**, and Reactome **(C)** annotation for DEGs. Bars represent the number of DEGs involved in the corresponding pathway. After YJ016 infection, the number of DEGs in the immune-related pathways in the Pair group was significantly higher than that in the EtOH group. The mean differences of three different methods were GO (45.69, *P* = 0.0003), KEGG (21.92, *P* < 0.0001), and Reactome (-31.33, *P* = 0.0082), respectively.

We selected characteristic pathways in signal transduction, cell proliferation and differentiation, chemotaxis and migration, and immune response to compare the functional changes of the EtOH and Pair groups after YJ016 infection (**Figure 3**), the detailed data was provided in Document S2. The GO annotation results (**Figure 3A**) showed that the number of DEGs related to these pathways in the Pair group was about three times more than that of the EtOH group in the liver, and 10 times in the spleen. KEGG and Reactome annotation results were consistent with GO annotation (**Figures 3B, C****)**.

Neutrophils play a central role in the early defense of most bacterial infections. In response to infection, they leave the circulation and migrate toward inflammatory lesions (32). Neutrophils can secrete cytokines and other inflammatory mediators through degranulation, and present antigens through MHC II to stimulate T cells (33). The number of DEGs related to neutrophil degranulation in the Pair group was three times more than that of the EtOH group (**Figure 3C**, 95 vs 26), which indicated that neutrophils might not function normally in ALD mice. The neutrophil degranulation dysfunction may affect the cytokines secretion, antigen presentation, and T cell function, which may lead to an impaired differentiation of other cells. The number of DEGs related to leukocyte differentiation (**Figure 3A**, 120 vs 34 in the liver, 81 vs 11 in the spleen) and Th17 cell differentiation (**Figure 3B**, 31 vs 9) in the Pair group was 3–7 times more than that of the EtOH group, which suggested that the differentiation of leukocytes was blocked, and the expression of immune molecules was abnormal in ALD mice. Leukocyte differentiation antigens are involved in recognizing and capturing antigens and can promote the interaction between immune cells and antigens, immune molecules, or immune cells (34). Leukocyte differentiation dysfunction can affect the exchange of information between immune cells. The number of DEGs related to the immune response-regulating signaling pathway (**Figure 3A**, 84 vs 21) and chemokine signaling pathway (**Figure 3B**, 42 vs 12), signaling by Rho GTPases (**Figure 3C**, 71 vs 2) in the Pair group was 3–35 times more than that of the EtOH group. Chemokine signals are transduced by the chemokine receptors (G protein coupled receptors) expressed on immune cells, and various members of GTPases are involved in this process (35). An effective inflammatory immune response first requires the recruitment of immune cells (such as neutrophils, cytotoxic T cells, and dendritic cells, etc.) to the inflammation site, and then identifying the pathogens through specific signaling pathways (such as NOD-like receptor signaling pathway, Toll-like receptor signaling pathway, etc.). After passing through the relevant signal pathways, the activated immune cells can phagocytose bacteria and release cytokines to promote the elimination of bacteria. The chemokine signal transduction pathway and immune regulation function incomplete will affect the migration and function of leukocytes. The number of DEGs related to chemotaxis (**Figure 3A**, 91 vs 17), leukocyte transendothelial migration (**Figure 3B**, 40 vs 7) in the Pair group was about five times more than that of the EtOH group, which suggested that the leukocytes could not migrate to the inflammation site normally in ALD mice. The migration of leukocytes from the blood to the tissues is crucial in dealing with bacterial infections (32), leukocyte migration dysfunction will directly lead to a weak immune response to bacteria. The number of DEGs related to the response to the bacterium, leukocyte mediated immunity (**Figure 3A**, 66 vs 19) in the Pair group was three times more than that of the EtOH group.

The degranulation of neutrophils, leukocyte differentiation, and chemotaxis can affect each other, and jointly promote the immune response and the elimination of pathogens. However, compared with the Pair group, these function-related DEGs were rare in ALD mice, suggesting that immune function might be impaired and bacterial clearance may be affected. Therefore, we subsequently determined the specific gene expression level and bacterial load to verify the results of the transcriptomics.

### DEGs Between EtOH_Y and Pair_Y

**Figure 4:**
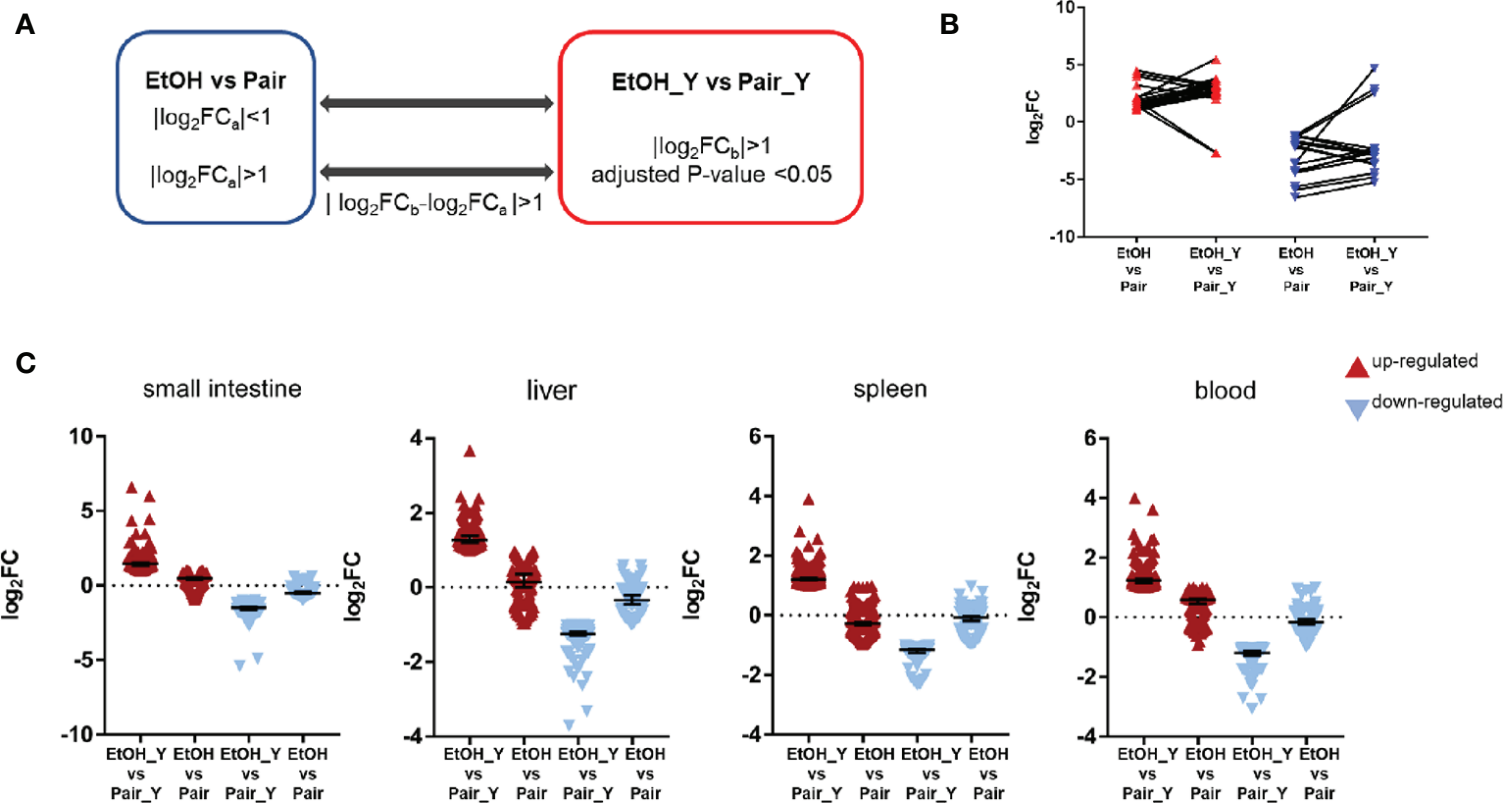
Differential expression analysis between EtOH_Y and Pair_Y. **(A)** Method for selecting the upregulated and downregulated genes in EtOH_Y compared with Pair_Y. The statistical parameters used as thresholds were: (1) |log_2_FC_a_| < 1 in control mice and |log_2_FC_b_| > 1, adjusted *P*-value < 0.05 in the case of YJ016 infection (2) |log_2_FC_a_| > 1 in control mice, |log_2_FC_b_| > 1, adjusted *P*-value < 0.05 and D values (|log_2_FC_b_-log_2_FC_a_ |) > 1 in the case of YJ016 infection. **(B)** The log_2_FC of DEGs selected by method A1. **(C)** The log_2_FC of DEGs selected by method A2.

In order to further analyze the DEGs after YJ016 infection, we compared the DEGs between EtOH_Y and Pair_Y (**Figure 4A**). Some genes in the EtOH and Pair groups did not change with LBS gavage, but their expression increased or decreased significantly with YJ016 gavage (**Figure 4B**). The number of up- and downregulated genes, respectively, were 119 and 74 (small intestine), 127 and 86(liver), 219 and 99(spleen), and 133 and 77 (blood). We also looked for genes whose expression levels differed between the EtOH and Pair groups, with D values (|log_2_EtOH_Y vs Pair_Y-log_2_EtOH vs Pair|) > 1 after YJ016 infection (**Figure 4C**).

The function of the characteristic genes is shown in **Table 1**, the detailed data was provided in **Supplementary Table 3**. In the small intestine, *Cma2* and *Mcpt9* genes were upregulated in the small intestine in the EtOH group, and their levels increased further after infection with YJ016. The mast cell protease plays an important role in the inflammatory response and may affect the permeability of the intestine and vascular endothelium (36). In the process of *V. vulnificus* infection, TLR 5 participate in the recognition of different bacterial components and play important roles in the immune response (37). In blood, the TLR5 expression level in ALD mice was significantly lower than that of the Pair group after infection. It may affect the downstream signal pathway, which is consistent with the results of the enrichment analysis (**Figures 3B, C**).Genes coding heat shock protein 1A and 1B in the blood of the EtOH group were upregulated, suggesting that ALD mice were in a stress state after infection (38).

**Table 1:** Several characteristic DEGs between EtOH_Y and Pair_Y.

Tissue	Gene name	log_2_FoldChange		D-value	Description
		EtOH_Y vs Pair_Y	EtOH vs Pair		
small intestine	*Nkx2-9*	-5.40	-0.12	-5.28	NK2 homeobox 9
	*mt-Tc*	-2.71	0.09	-2.80	mitochondrially encoded tRNA cysteine
	*Sycp3*	2.45	-0.70	3.15	synaptonemal complex protein 3
	*Rgs13*	1.69	-0.92	2.62	regulator of G-protein signaling 13
	*Cyp1a1*	4.67	-3.58	8.25	CYP450, subfamily 1a, polypeptide 1
	*Cma2*	3.58	1.46	2.12	chymase 2, mast cell
	*Mcpt9*	3.20	1.21	1.99	mast cell protease 9
liver	*Derl3*	-3.71	0.45	-4.16	Der1-like domain family, member 3
	*Zfp966*	3.68	0.40	3.28	zinc finger protein 966
	*Syngr1*	2.66	3.99	-1.34	synaptogyrin 1
spleen	*Sln*	-2.20	0.97	-3.17	sarcolipin
	*Ccne2*	1.71	-0.90	2.60	cyclin E2
	*Arhgdig*	2.37	-1.33	3.69	Rho GDP dissociation inhibitor gamma
	*Hist4h4*	2.07	-1.30	3.37	histone cluster 4, H4
blood	*Tlr5*	-1.81	0.91	-2.72	toll-like receptor 5
	*Mrgpra2b*	-2.22	0.24	-2.46	MAS-related GPR, member A2B
	*Hspa1b*	2.80	0.08	2.72	heat shock protein 1B
	*Melk*	2.92	1.58	1.34	maternal embryonic leucine zipper kinase
	*Hspa1a*	2.59	1.23	1.36	heat shock protein 1A

### Quantitative Real-Time PCR (RT-PCR) Analysis of DEGs

**Figure 5:**
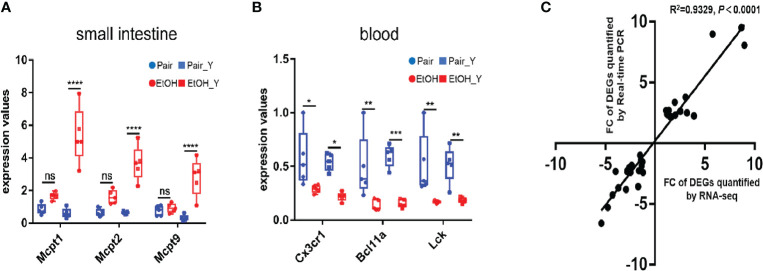
RT-PCR analysis of DEGs. **(A)** RT-PCR results of DEGs in the small intestine. The *Mcpt1*, *Mcpt2*, and *Mcpt9* genes had higher expression levels in EtOH_Y than in Pair_Y (n = 5). **(B)** RT-PCR results of DEGs in the blood. The *Cx3cr1*, *Bcl11a*, and *Lck* genes had lower expression levels in EtOH_Y than Pair_Y (n = 5). **(C)** The correlations were measured by dispersing the fold changes of DEGs between RNA-Seq and RT-PCR. (*P < 0.05, **P < 0.01, ***P < 0.001, ****P < 0.0001). ns, no significant.

After the analysis of DEGs, we verified the results of transcriptomics at the two levels of gene expression and phenotype. The RT-PCR analysis (**Figure 5**, with detailed data in Document S3) showed that in the small intestine, the *Mcpt1*, *Mcpt2*, and *Mcpt9* genes encoding mast cell proteases were upregulated in EtOH_Y compared with Pair_Y; in the blood, the genes encoding the chemokine (C-X3-C motif) receptor 1, B cell CLL/lymphoma 11A (zinc finger protein), and lymphocyte protein tyrosine kinase were downregulated in EtOH_Y compared with Pair_Y. A high correlation (Pearson’s *R^2^* = 0.9329) was found between the RT-PCR and RNA-Seq results (**Figure 5C**), which showed that the results of transcriptomic sequencing were reliable.

### Post-Infection Blood Differences Between the EtOH and Pair Groups

**Figure 6:**
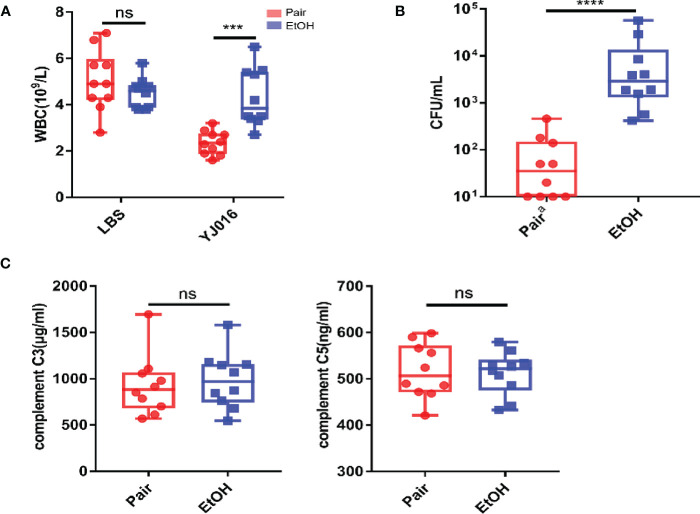
Blood bacterial loads, blood counts, plasma complement. **(A)** Blood bacterial loads in the Pair and EtOH groups (n = 10). **(B)** White blood cell counts in the Pair and EtOH groups (n = 10). The number of white blood cells decreased significantly in the Pair group compared with the EtOH group post-infection. **(C)** Plasma complement C3 and C5 concentrations in the Pair and EtOH groups (n = 10). There was no statistically significant difference in complement C3 and C5 between the Pair and EtOH groups. (ns. *P* > 0.05, ***P < 0.001, ****P < 0.0001).

An interesting finding in the phenotypic verification is that the blood bacterial load (CFU/ml) of 4 out of 10 mice in the pair group was below the lower limit of detection at 12 hours post infection, others are less than 10^3^; the blood bacterial load (CFU/ml) was more than 10^3^ in 6 mice and even more than 10^4^ in 2 mice in the EtOH group (**Figure 6A**). The bacterial load of the EtOH group was about 100 times higher than that of the Pair group, which might be related to the increased intestinal permeability and weaker immune response in ALD mice. The blood counts showed that the number of WBC in the peripheral blood of the Pair group significantly decreased after infection (**Figure 6B**). It is consistent with the transcriptomics results of chemotaxis and migration (**Figure 3**). Plasma complement (C3, C5) content in mice with LBS gavage revealed no significant difference between the EtOH and Pair groups (**Figure 6C**). This indicates that the difference in the blood bacterial load was probably not caused by complement.

### Cytokines Differences Between the EtOH and Pair Groups

**Figure 7:**
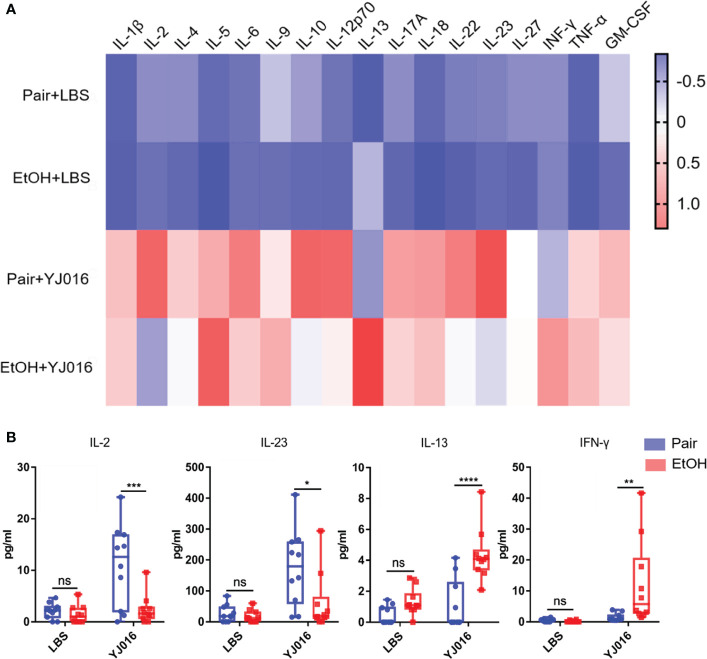
Cytokine concentrations in the Pair and EtOH groups. **(A)**. The values of cytokines concentrations were standardized by z-score, and the means were used to draw the heat map with red indicating a higher concentration and blue indicating a lower concentration (n = 10). **(B)** Cytokines with significantly different concentrations in the EtOH and Pair groups after YJ016 infection (n = 10). The plasma levels of IL-2 and IL-23 in the Pair group were higher than those in the EtOH group; IL-13 and IFN-γ were lower in the EtOH group than those in the Pair group. (*P < 0.05, **P < 0.01, ***P < 0.001, ****P < 0.0001).

The differences of cytokines between the EtOH and Pair groups post infection were analyzed in the present study (**Figure 7A**). The results showed that after infection with YJ016, the plasma levels of IL-2 and IL-23 in the Pair group were higher than those in the EtOH group; IL-13 and IFN-γ were lower in the EtOH group than in the Pair group (**Figure 7B**). IL-2 has been shown to be an essential T cell growth factor for the proliferation of T cells (39). IL-23 is a pro-inflammatory cytokine which induces the proliferation and differentiation of Th17 cells (40). IFN-γ can inhibit the proliferation of Th17 cells driven by IL-23 (40). IL-13 can inhibit the production of inflammatory cytokines and chemokines, and indirectly affect the activation of T cells by other immune cells (41). These results showed that after infection, ALD mice might have an immune cell dysfunction and a certain degree of immunosuppression, which is consistent with the decrease in the number of DEGs and the increase in the bacterial load in transcriptomics. But the reasons for immunosuppression and specific function of the immune cells need further study.

## Discussion

The mortality rate for sepsis caused by *V. vulnificus* infection is about 50%, of which, the morbidity and mortality in ALD patients are higher than others (2, 11). Studies have shown that the immune function of patients with ALD is generally impaired (16). However, after infection with *V. vulnificus*, whether the immune response of ALD patients is different from others remains unclear. Exploring the difference in the immune response between healthy individuals and ALD patients and its impact on the outcome of infection is crucial to understanding the reasons why ALD patients are susceptible to *V. vulnificus* infection. The present research found that many DEGs are immune response-related, and possibly play important roles in the susceptibility of ALD patients to *V. vulnificus* infection.

In the face of infection, the immune system will first recognize microorganisms, then promote the proliferation and differentiation of immune cells through signal transduction, and secrete cytokines to induce leukocytes to migrate to the location of the pathogen (42). The enrichment analysis results showed that the number of DEGs related to neutrophil degranulation, leukocyte differentiation, and chemotaxis in ALD mice were greatly less than those in the Pair group. The same difference was reflected in signal transduction pathways that affect pathogen recognition (43) [e.g., NOD-like receptor and Toll-like receptor signaling pathway (**Figure 3B**)]; cell differentiation (44) [e.g., the Wnt signaling pathway (**Figure 3A**), PI3K-Akt signaling pathway (**Figure 3B**)]; chemotaxis (45) [e.g., the Rap1 signaling pathway (**Figure 3C**)]. Previous studies have found that compared with healthy people, the activity of blood neutrophils in ALD patients is reduced, which favors *V. vulnificus* survival in the blood. Our research suggests that in addition to neutrophil activity, there may also be obstacles to the signaling transduction pathways in ALD mice, which may affect the immune response and the elimination of bacteria *V. vulnificus*. Besides, the NLR signaling is crucial for *V. vulnificus* infection, the absence of NLRP3 in macrophages impaired *V. vulnificus*-induced phagosome acidification and phagolysosome formation, leading to a reduction of intracellular bacterial clearance (46, 47). In our study, we found that the number of DEGs related to the NLR signaling pathway was reduced in ALD mice. It may be related to the immune dysfunction caused by ALD, and the specific reasons need to be further studied.

In addition to the difference in the number of DEGs with signal transduction and immune response, there were also differences in the gene expression levels. In the small intestine of ALD mice, genes encoding mast cell proteases, related to intestinal wall and blood vessel permeability, were upregulated (36) (**Figure 4A**), suggesting that YJ016 can enter the blood more easily after infection in ALD mice. In the blood of ALD mice, genes encoding chemokine (C-X3-C motif) receptor 1 and proteins of immune cells were downregulated (**Figure 4B**), implying an abnormal chemotaxis and function of immune cells in the blood (48). The bacterial load of ALD mice increased by about 100 times to the Pair group, while the number of peripheral blood leukocytes before and after infection did not change significantly, indicating that there were barriers to bacterial clearance and leukocyte chemotaxis in ALD mice.

The cytokine determination suggests that there may be obstacles to T cell function in ALD mice. IL-2 is mainly produced by activated T cells which can stimulate the proliferation of T cells and promote the production of cytokines (39). IL-2 increased significantly in the Pair group post-infection and was higher than the ALD mice (**Figure 7B**). The obstacle of T cell activation might be the reason for the decreased secretion of IL-2, and the low level of IL-2 can act against T cells, further affecting their activation (39). Besides, IL-23 that promotes T cell proliferation (40) decreased, and the concentration of cytokines (IL-13, IFN-γ) that inhibit T cell function increased (41). The combined effect of the two factors have blocked the differentiation of mouse T cells in the EtOH group. Other inflammatory factors such as IL-1β, IL-6, and TNF-α were not significantly different (**Figure 7A**), while the number of DEGs in the TNF-related signaling pathway was significantly different (**Figure 3**). Transcriptomics results showed that *Tnfrsf1a* encoding TNF-α receptors were upregulated in the liver and spleen of mice in the Pair group after infection, suggesting that the expression level of the receptors can also affect the immune response. The relationship between the downregulation of receptor expression and the decrease in the number of DEGs involved in immune response in ALD mice remains to be clarified.

In fact, most severe infections of *V. vulnificus* occur in patients with underlying conditions resulting in primarily alcohol-associated liver cirrhosis or immuno-compromised males, but it does not cause severe illness in healthy individuals (11). Although there have been many studies on *V. vulnificus*, few animal models that mimic human infection were used to elucidate the underlying mechanisms that contribute to acute infections by *V. vulnificus* in liver disease patient. To sum up, this is the first systematic study on *V. vulnificus* infection using an ALD murine model. We found that after *V. vulnificus* gavage, compared with the Pair group, the number of DEGs related to immune function decreased in the liver, spleen, and blood in ALD mice, possibly making them susceptible to infection with *V. vulnificus*. This study is a valuable resource for describing mice with ALD after infection with *V. vulnificus*, and it provides potential targets for further investigation in the context of treatment options for *V. vulnificus* sepsis.

## Data Availability Statement

The datasets presented in this study can be found in online repositories. The names of the repository/repositories and accession number(s) can be found in the article/**Supplementary Material**.

## Ethics Statement

The animal study was reviewed and approved by The Ethics Committee of Academy of Military Medical Sciences.

## Author Contributions

Z-HF, YY, and J-LW conceived and designed the experiments. Z-HF, S-QL, X-RB, and J-HX performed the animal experiments. Z-HF and S-QL performed the sample collection, blood bacterial load, and cytokine analysis. Z-HF, YY, and J-XZ performed the sequencing data and bioinformatics analysis. BN, Z-bL, W-WX, LK, SG, JW, and J-XL contributed the reagents/materials/analysis tools. Z-HF, YY, and J-LW wrote the paper. All authors contributed to the article and approved the submitted version.

## Conflict of Interest

The authors declare that the research was conducted in the absence of any commercial or financial relationships that could be construed as a potential conflict of interest.

## Publisher’s Note

All claims expressed in this article are solely those of the authors and do not necessarily represent those of their affiliated organizations, or those of the publisher, the editors and the reviewers. Any product that may be evaluated in this article, or claim that may be made by its manufacturer, is not guaranteed or endorsed by the publisher.

## Supplementary Material

Supplementary Figure 1 Cytokine concentrations in different periods. **(A)**. Cytokine concentrations in plasma 9 hours and 5 days post gavage (n = 10). The concentrations of cytokines were standardized by z-score, with red indicating higher concentration and blue indicating lower concentration. **(B, C)**. The concentration of IL-18 and IL-4 (ns P>0.05, **P<0.01, ****P<0.0001).
