# Supplementary figures and images for: Analysis of Gene Expression Profiles, Cytokines, and Bacterial Loads Relevant to Alcoholic Liver Disease Mice Infected With V. vulnificus
Source: Front Immunol. 2021 Aug 20;12:695491. doi: 10.3389/fimmu.2021.695491 (PMC8417779; doi:10.3389/fimmu.2021.695491)

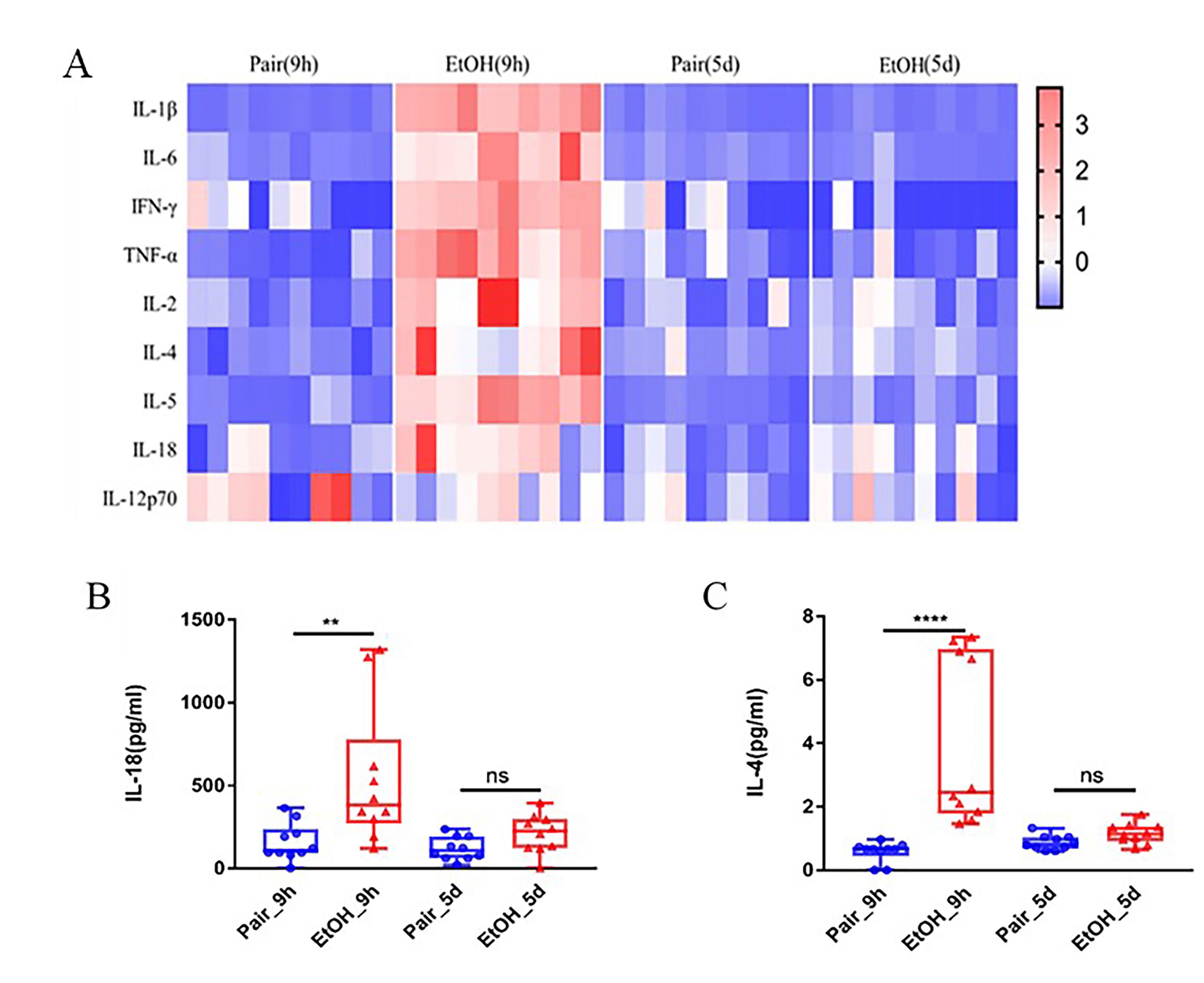

Supplement: Supplementary Figure 1 — Cytokine concentrations in different periods. (A). Cytokine concentrations in plasma 9 hours and 5 days post gavage (n = 10). The concentrations of cytokines were standardized by z-score, with red indicating higher concentration and blue indicating lower concentration. (B, C). The concentration of IL-18 and IL-4 (ns P>0.05, **P<0.01, ****P<0.0001). [file Image_1.jpeg]
